# Minorities and foreign born are disproportionately affected by injuries due to violence: an analysis based on a National Trauma Registry 2008–2017

**DOI:** 10.1186/s13584-019-0297-5

**Published:** 2019-03-07

**Authors:** Abebe Tiruneh, Irina Radomislensky, Israel Trauma Group, H. Bahouth, A. Becker, A. Hadary, I. Jeroukhimov, M. Karawani, B. Kessel, Y. Klein, G. Lin, O. Merin, B. Miklush, Y. Mnouskin, A. Rivkind, G. Shaked, G. Sibak, D. Soffer, M. Stein, M. Wais, H. Pharan, I. Garbetzev, Kobi Peleg, Maya Siman-Tov

**Affiliations:** 1 Israel National Center for Trauma and Emergency Medicine Research, Gertner Institute for Epidemiology and Health Policy Research, Sheba Medical Center, Tel-Hashomer, 52621 Ramat Gan, Israel; 2 Department of Disaster Management, School of Public Health, Tel Aviv University, Tel Aviv, Israel

**Keywords:** Ethnicity, Foreign born, Injury, Violence, Hospitalization

## Abstract

**Background:**

Populations of different ethnicity and country of origin living in the same country may possess particular features of violence-related injuries. This study aims to compare violence-related injury characteristics and circumstances, hospital resource utilization and in-hospital mortality among the major ethnic groups in Israel.

**Methods:**

A study based on the Israeli National Trauma Registry database of patients hospitalized due to violence-related injuries between 2008 and 2017. Data included demographic, injury and hospitalization characteristics and in-hospital mortality. Statistical analysis included *χ*^2^-test and multiple logistic regression.

**Results:**

During the study period, 16,151 violence related-hospitalizations were reported, of which; 46.1% were Arab Israelis (AI), 3.2% were Israelis born in Ethiopia (IBE), 12.7% were Israelis born in the former Soviet Union (IBFSU) and 38.0% were all other Israelis (AOI). The proportion of violence-related hospitalizations among AI, IBE and IBFSU was greater than their respective proportion in the Israeli population. In comparison to the other groups, stab injuries were significantly greater among IBE (30% vs 39%); unarmed brawl-related injuries were greater among IBFSU (22–41% vs 49%) and firearm injuries were greatest among AI (2–8% vs 23%). These differences in violence mechanism persisted even after accounting for age, gender, injury place and time differences. The foreign born groups had higher rates for injuries sustained on the street/road (58% for IBE, 54% for IBFSU vs 46% for AI and AOI, each), with IBE also showing higher rates for weekend and weeknight injuries compared to the other groups (83% vs 71–75%). IBE were more likely to suffer from severe and critical injuries (19% vs 12–16%), to be admitted to the intensive care unit (17% vs 9–11%) and to have prolonged hospital stays of seven days or more (20% vs 16–17%), with no significant difference in in-hospital mortality between the comparison groups.

**Conclusions:**

Characteristics of violence-related casualties differed significantly among diverse ethnic populations living in the same country. Each population group showed specific attributes regarding injury mechanism, circumstances, severity and hospital utilization. Violence prevention programs should be culturally adapted and take into account ethnicity and country of origin of the target population.

## Introduction

Violence undermines people’s health and some groups are more affected by violence than others. Race and ethnicity are among the factors that distinguish population groups most at risk of violence, with ethnic minorities being disproportionately affected [1–7]. Studies in several countries have shown that appreciable portions of the gaps in exposure to violence and its consequences between ethnic groups can be accounted for by family background, individual differences, and neighborhood factors [8–10]. Ethnic differences associated with violence are also linked to sociocultural (such as parenting styles and masculinity norms) and immigration factors, such as social disintegration [1, 11–13].

According to a report by the World Health Organization (WHO) Region for Europe, in comparison to other European countries, Israel ranked 9th in homicide among people aged 10–29 years at a rate of 4.30 per 100,000 population [1]. A report produced by the Israel Ministry of Public Security in collaboration with the Central Bureau of Statistics (CBS) showed that there were approximately 620,000 annual incidents of violence in Israel between 2003 and 2010; with an upward trend between 2011 and 2012, particularly for serious violent offenses [14]. In addition, disparities in violence-related injuries between ethnic groups in Israel have been reported [15, 16]. The risk of being hospitalized for violence-related injuries was higher among non-Jewish Israeli residents compared with Jewish Israelis [17].

The Israel society is composed of two main ethnic populations: Jews and Arabs, comprising 75.1 and 20.6%, respectively, according to the Israeli CBS data for the years 2008–2017 [18]. Jews and Arabs are the two major distinct ethnic groups who may experience different risks of exposure, vulnerability and outcomes from injuries as a result of violence, because these populations differ in many ways, including socio-cultural differences [17, 19–22]. Among the Jewish population, there were two major waves of immigration to Israel in the 1990’s. These immigrant groups included Ethiopian Jewish immigrants and immigrants from the former Soviet Union, 1.0 and 7.9%, respectively, of the general Israel population, according to the Israeli CBS, 2008–2017 [23]. It is important to note that the proportion of Ethiopian and former Soviet Union immigrants presented above does not include descendants born in Israel. These population groups faced the challenges of reestablishing life in a new country [24, 25], which may be associated with increased susceptibility to violence and related injuries. In comparison with Arab and Jewish born Israelis, studying violence-related injuries among these immigrant populations can help understand the potential impact of immigration on violence-related injuries. The Ethiopian community and Arabs are amongst the ethnic groups in Israeli society with the lowest socioeconomic status (SES). These two population groups are comprised of large families and a relatively young population. The majority of Arabs live in towns and villages where most of the residents are Arabs. While the majority of Ethiopian Israelis reside in Jewish cities, they often live in segregated neighborhoods with large numbers of Ethiopian-Israeli residents [25–29]. These sociodemographic characteristics may be associated with differential injury risks from violence [30–32]. It should be noted that every Israeli resident, regardless of ethnicity, gender or country of origin is entitled to health care services under the National Health Insurance Law. Equally important, fees are not prerequisite for receiving care [33].

In this paper we sought to examine ethnic differences associated with violence-related injuries. In Israel, characteristics and circumstances of violence-related hospitalizations, hospital resource utilization and in-hospital mortality in relation to ethnicity and country of origin have not been well documented. The outcomes from this study provide evidence based data which can enable policy makers to focus on intervention programs for population at high risk.

## Methods

This study was based on the Israeli National Trauma Registry (ITR) database of patients hospitalized for injuries due to violence between January 1, 2008 and December 31, 2017. The ITR is an extensive database of hospitalized trauma patients, providing a broad geographic and demographic coverage in the country [15, 16]. All the six Level Ι Trauma Centers and 14 Level ΙΙ Trauma Centers participated in the ITR during the study period. There were five hospitals that did not participate in the ITR during the study period, representing only 5% of the total hospitalized trauma patients in Israel. Included in the ITR are injured trauma patients with an International Classification of Diseases, Ninth Revision (ICD-9-CM) diagnosis code of 800–959.9 who were hospitalized, died in the hospital (including deaths in the emergency department) or were transferred to or from another hospital for admission. The ITR does not include patients who died at the scene of the event, on the way to hospital or on arrival; who were discharged following treatment in the emergency department; or who were hospitalized 72 h or more after the event. Data reported in the registry are recorded by trained trauma registrars at each trauma center under the supervision of a trauma director and trauma coordinator. Electronic files are transferred daily to the National Center for Trauma and Emergency Medicine Research where quality assurance is carried out prior to the data being analyzed. Unclear or erroneous data are referred back to the trauma centers for clarification or completion. The data in the ITR are anonymous and there is no way to identify patients; the study received the approval of the Sheba Medical Center Institutional Review Board (IRB) (Number 5138–18 SMC). From this comprehensive database, this study focused on analyzing violence-related injuries, identified using codes for external cause (E-code) of injury ‘E960.0’-'E968–.9′. Self-inflicted injuries and injuries from terror attacks and war were not included. In order to avoid double counting for transferred patients, the data from their last hospitalization was used. Since the study focused only on Israeli residents, Non-Israeli Arab residents of East Jerusalem (311, 1.8%), foreign workers (544, 3.1%), tourists (141, 0.8%) and unidentified casualties (90, 0.5%) were excluded from the analysis. These groups were excluded on the assumption that hospitalization characteristics and outcomes at hospital discharge of non-citizens may differ from those of citizens primarily because of insurance coverage differences.

The data measured included demographic characteristics (age, gender, ethnicity, country of birth); injury characteristics (mechanism of violence, type of injury, injury severity, injured body region, traumatic brain injury); injury setting and timing of hospital arrival; hospital resource utilization (treatment in trauma resuscitation unit, undergoing surgery, admission to intensive care unit and length of hospital stay) and in-hospital mortality. Age in years was divided into five categories: 0–14, 15–24, 25–44, 45–64, 65+. The Injury Severity Score (ISS) was used to quantify injury severity, which was classified into two groups: 1–14 (mild and moderate injuries) and 16–75 (severe and critical injuries), derived from its four categories [34, 35]. Mechanism of violence was categorized as: firearm, stabbing, assault with object, unarmed brawl and other. Type of injury was categorized as penetrating versus non-penetrating and place of injury was classified as injury event occurring on the street/road, at home, in public (which included commercial/leisure places, playing/tour places or sport centers) and other (which included residential institution, workplace, school, army base, farm land, sea/lake, air and others). Time of hospital arrival was defined as follows: weekday as daytime from 06:00 to 18:59 Sunday through Thursday; weeknight as night hours from 19:00 to 05:59 from Sunday19:00 to Thursday 05:59; and weekend as any time between Thursday at 19:00 and Sunday at 05:59. It is important to note that in Israel Sunday is a work day and Friday is not a work day for government offices. For this study’s purpose, time of patient presentation to the hospital could be taken as a proxy for time of injury occurrence, as the country is of a relatively small surface area [36], and it is known for its advanced trauma system with short arrival times. Trauma centers are located throughout the country; enabling trauma casualties to receive rapid treatment in all parts of the country. Due to redistribution of ambulance dispatch centers and the use of geographic information systems and Global Positioning System, in recent year’s response times have been reduced [15, 16, 37–40]. The Abbreviated Injury Scale (AIS) codes and scores of nine body regions (head, face, neck, thorax, abdomen, spine, upper extremity, lower extremity and external) were used for recognizing the injured body regions. Traumatic brain injury was defined as any recorded evidence of brain injury in accordance with the AIS 1990 Revision Manual [41]. Number of injured body regions was categorized as single (when only one body region was injured) and multiple (when more than one region was injured). Treatment in trauma resuscitation unit (yes/no), admission to intensive care unit (yes/no), undergoing surgery (yes/ no), length of hospital stay (> 7 days or < 7 days) and in-hospital mortality (yes/no) were coded as binary variables.

Analyses were performed using SAS statistical software version 9.4 (SAS Institute, Cary. NC, USA). Missing data for each variable entered into the analyses were less than 0.1%. Descriptive data were compared by χ^2^-test and multiple logistic regression models were developed to compare the mechanisms of violence as well as injury severity, injury type and requirement of admission to intensive care unit between the ethnic groups. A *p*-value of < 0.05 was considered statistically significant.

## Results

### Study population

**Fig. 1:**
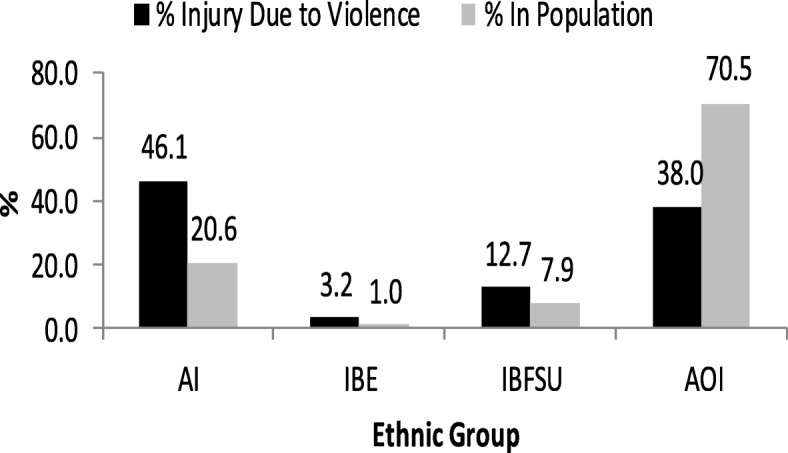
Distribution of violence-related hospitalizations by ethnic group and distribution of ethnic group in the total population, Ten-year period 2008–2017. Abbreviations: AI-Arab Israelis, IBE- Israelis Born in Ethiopia, IBFSU-Israelis Born in former Soviet Union, AOI-All other Israelis. *The proportion of each ethnic group in the general population shown in the figure is based on the data from the Israel Central Bureau of Statistics, 2009–2018 [18, 23]. Note that the average population was taken for the years 2008 to 2017 for each population group to calculate its proportion in the general population

A total of 16,151 hospital admissions due to violence-related injuries were included in this study. Of them, 7445 (46.1%) were AI, 522 (3.2%) were IBE, 2044 (12.7%) were IBFSU and 6140 (38.0%) were AOI. These results indicate that the distribution of AI, IBE and IBFSU among violence-related hospitalizations was higher than their respective proportion in the general Israeli population, (20.6% for AI, 1.0% for IBE, 7.9% for IBFSU and 70.5% for AOI [18, 23], Fig. 1).

### Demographics, injury characteristics and circumstances

As displayed in Table 1, age distribution varied within each comparison group with persons aged 15–44 years constituting the vast majority of violence-related casualties in all ethnic groups while the percentage was significantly greater among IBE (85%) and AI (79%) compared to IBFSU (71%) and AOI (69%). Males constituted the majority of the patients in all ethnic groups, IBE (92%), AI (90%), IBFSU (86%) and AOI (85%). The populations significantly varied by place and time of injury event; with IBE having highest rates for injuries sustained on the street/road (58, 47, 54, 46% respectively for IBE, AI, IBFSU and AOI) and for injuries occurring during the weekend/weeknight (83, 75, 71 and 71% respectively for IBE, AI, IBFSU and AOI), see Table 1.

**Table 1:** Violence-related hospitalizations 2008–2017: Demographic and injury event characteristics by ethnic group

Variable	Total (*N* = 16,151)	AI (46.1%)	IBE (3.2%)	IBFSU (12.7%)	AOI (38.0%)	*P value* ^a^
Age, %						< 0.0001
0–14	9.1	9.5	4.0	1.6	11.6	
15–24	38.5	41.9	40.4	25.5	38.4	
25–44	35.7	36.8	44.5	45.7	30.2	
45–64	13.0	10.4	8.2	20.1	14.2	
65+	3.7	1.4	2.9	7.1	5.5	
Gender (Male), %	88.0	90.3	91.6	86.1	85.5	< 0.0001
Injury setting, %						< 0.0001
Street	48.1	47.5	58.4	54.4	45.8	
Home	11.3	10.1	11.3	12.9	12.3	
Public	8.8	4.6	12.8	12.9	12.3	
Other	31.8	37.8	17.4	19.8	29.6	
Hours of a day and days of a week, %						< 0.0001
Weeknight/Weekend	73.9	75.5	82.8	74.7	70.9	
Weekday	26.1	24.5	17.2	25.3	29.1	

Abbreviations: *AI* Arab Israelis, *IBE* Israelis Born in Ethiopia, *IBFSU* Israelis Born in former Soviet Union, *AOI* All other Israelis

^a^*P-values* were determined by *X*^*2*^

**Fig. 2:**
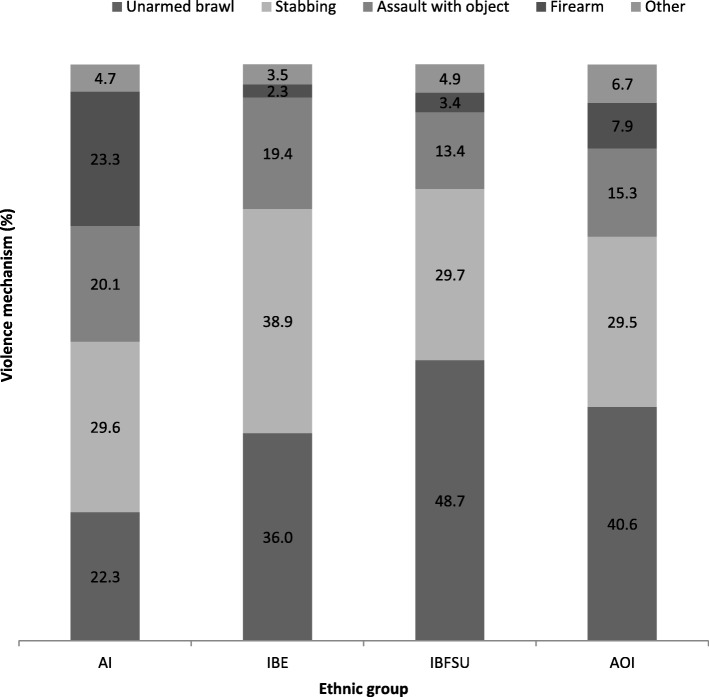
Violence mechanism by ethnic group. Abbreviations: AI-Arab Israelis, IBE- Israelis Born in Ethiopia, IBFSU-Israelis Born in former Soviet Union, AOI-All other Israelis. *P* < 0.0001, determined by *X2* test.

The most frequent mechanisms of violence-related injuries were unarmed brawls and stabbing, which together accounted for 63% of all violence-related hospital admissions. As shown in Fig. 2, the distribution by the violence mechanism revealed a significantly greater proportion of injuries from firearms among AI (23%) in comparison to IBE (2%), IBFSU (3%) and AOI (8%). Stabbing-related injuries were significantly more common among IBE (39%) compared with the other groups (30%), while unarmed brawl injuries were most frequent among IBFSU (49% compared to 22–41% among the other population groups). Penetrating injuries were significantly more prevalent among AI (57%) compared with the other ethnic groups (38–46%). Injured body region also varied between the ethnic populations, with head and face injuries being more prevalent among the immigrant populations (IBE and IBFSU), and injuries to extremities were more common among AI followed by AOI. Injury severity varied by ethnic group, with a greater percentage of IBE and IBFSU suffering from severe and critical, injuries Table 2.

**Table 2:** Violence-related hospitalizations 2008–2017: Injured body region, hospital resource utilization and in-hospital mortality by ethnic group

Variable	Total (N = 16,151)	AI (46.1%)	IBE (3.2%)	IBFSU (12.7%)	AOI (38.0%)	p-value^a^
Injury severity score (ISS16+)	13.4	13.4	19.4	15.6	12.1	< 0.0001
Penetrating injury	48.8	57.1	46.0	38.5	42.3	< 0.0001
Injured body region^b^						
Head	30.5	29.2	38.7	37.6	29.0	< 0.0001
Face	28.0	22.7	36.2	40.3	29.5	< 0.0001
Neck	4.1	3.4	6.5	4.5	4.7	< 0.0001
Thorax	22.1	22.6	20.5	22.3	21.6	NS
Abdomen	24.1	24.5	24.7	21.6	24.3	0.0496
Spine	2.0	2.0	0.8	2.1	2.0	NS
Upper extremity	20.4	22.5	15.1	17.1	19.5	< 0.0001
Lower extremity	21.1	26.3	10.1	13.8	18.1	< 0.0001
External	1.8	1.4	0.8	1.7	2.5	< 0.0001
Multiple Injuries	38.1	38.3	39.3	42.9	36.1	< 0.0001
Traumatic brain injury	8.9	7.8	16.5	13.9	7.9	< 0.0001
Treated in trauma resuscitation unit	37.0	41.0	40.8	33.5	33.0	< 0.0001
Admission to ICU	10.2	10.7	17.4	9.2	9.3	< 0.0001
Underwent surgery	33.1	33.3	32.6	33.1	32.8	NS
Hospital LOS (7 days or more)	16.3	16.7	19.9	16.3	15.6	< 0.05
Hospital LOS (14 days or more)	5.3	5.6	6.5	4.4	5.2	NS
Died in hospital	1.6	1.8	1.5	1.1	1.5	NS

Abbreviations: *AI* Arab Israelis, *IBE* Israelis Born in Ethiopia, *IBFSU* Israelis Born in former Soviet Union, *AOI* All other Israelis, *ICU* intensive care unit, *LOS* length of stay

^a^*P-values* were determined by *X*^*2*^ test (NS = non-significant, i.e. *P* > 0.05)

^b^Note that a patient could sustain injury to more than one body region

### Hospital resource utilization and in-hospital mortality

IBE had a significantly higher rate of admission to the intensive care unit (ICU) following violence-related injuries compared to the other ethnic groups (19% compared with 9–11%, respectively for IBE and other groups). IBE and AI had higher rates of being treated in the trauma resuscitation unit (41% for IBE and AI, compared with 33% for IBFSU and AOI, each). IBE had prolonged hospital stay of seven days or more compared with the other populations (20, 17, 16 and 16%, respectively for IBE, AI, IBFSU and AOI). There was no significant difference for undergoing surgery and in-hospital mortality between the ethnic groups (Table 2).

### Adjusted analysis

#### Mechanism of violence

According to estimates of logistic regression analyses adjusted for age, gender, place and time of injury event; in comparison to AOI, AI had a significantly greater risk of hospitalization due to injuries from firearm [OR (95% CI): 3.36 (3.01–3.75)] as well as assault with object [OR (95% CI): 1.40 (1.28–1.54)), and IBE had a greater risk for injuries from stabbing [OR (95% CI): 1.29 (1.07–1.56)] or assault with object [OR (95% CI): 1.41 (1.12–1.77)], while IBFSU had a higher risk for unarmed brawl-related injuries [OR (95% CI): 1.47 (1.32–1.63)], Table 3. In a separate logistic regression analyses adjusted for age, gender, place and time of injury event (not displayed in table), the risk of hospitalization due to firearm-related injuries was also significantly greater among AI in comparison to IBE [OR (95% CI): 14.64 (8.63–27.62)] and to IBFSU [OR (95% CI): 9.74 (7.66–12.60)]. A significantly greater risk for stab injuries was reported among IBE in comparison to AI [OR (95% CI):1.43 (1.18–1.72)] and to IBFSU [OR (95% CI): 1.31 (1.06–1.60)]; while IBFSU had a higher risk for unarmed brawl-related injuries than IBE [OR (95% CI): 0.67(0.55–0.82)] and AI [OR (95% CI): 0.31(0.28–0.35)]. The risk for injuries by assault with an object was greater in IBE [OR (95% CI): 1.55(1.20–1.99)] and AI [OR (95% CI): 1.54(1.34–1.78)] when compared with IBFSU, while there was no significant difference between IBFSU and AOI.

**Table 3:** Risk of hospitalization due to injury from a specific mechanism of violence among ethnic groups (N = 16,151)

Ethnic groups	OR(95% CI)^a^			
	Firearm	Stab	Assault with object	Unarmed brawl
AI vs AOI	3.36 (3.01–3.75)	0.91 (0.84–0.98)	1.40 (1.28–1.54)	0.46 (0.42–0.49)
IBE vs AOI	0.23 (0.12–0.39)	1.29 (1.07–1.56)	1.41 (1.12–1.77)	0.98 (0.81–1.19)
IBFSU vs AOI	0.34 (0.26–0.44)	0.99 (0.88–1.11)	0.91 (0.78–1.05)	1.47 (1.32–1.63)

Abbreviations: Abbreviations: *AI* Arab Israelis, *IBE* Israelis Born in Ethiopia, *IBFSU* Israelis Born in former Soviet Union, *AOI* All other Israelis, *OR* Odds ratio, *CI* Confidence interval

^a^Adjusted for age (categorized into five groups: 0–14, 15–24, 25–44, 45–64, 65+), gender, injury place (grouped in to: street, home, public and other) and time (classified in to two: weekend/weeknight and weekday). The reference group for the model of each mechanism was all other mechanisms of violence

#### Injury severity, injury type and admission to intensive care unit

In comparison to AOI, the immigrant groups (IBE and IBFSU) were significantly more likely to suffer from violence-related severe and critical injury after controlling for the effects of age, gender, violence mechanism and injury type. There was no significant difference in the risk of experiencing penetrating injury between the three population groups (AI, IBE or IBFSU) and AOI after accounting for age, gender and violence mechanism. The increased likelihood of being admitted to ICU among IBE versus AOI persisted even after controlling the effects of age, gender, ISS, violence mechanism and injury type (Table 4).

**Table 4:** Likelihood of violence-related severe and critical injury, penetrating injury and admission to intensive care unit among ethnic groups (*N* = 16,151)

Variable	OR (95% CI)		
	AI vs AOI	IBE vs AOI	IBFSU vs AOI
Severe and critical injury (ISS 16+)^a^	0.92 (0.83–1.02)	1.78 (1.40–2.25)	1.38 (1.19–1.59)
Penetrating injury^b^	0.99 (0.85–1.16)	0.86 (0.56–1.29)	1.07 (0.85–1.34)
Admission to ICU^c^	0.95 (0.83–1.09)	1.92 (1.42–2.58)	0.93 (0.76–1.14)

Abbreviations: *AI* Arab Israelis, *IBE* Israelis Born in Ethiopia, *IBFSU* Israelis Born in former Soviet Union, *AOI* All other Israelis, *OR* Odds ratio, *CI* Confidence interval, *ISS* Injury severity score, *ICU* Intensive care unit

^a^Adjusted for age, gender, violence mechanism and injury type

^b^Adjusted for age, gender and violence mechanism

^c^Adjusted for age, gender, ISS, violence mechanism and injury type

Note that age was categorized into five groups (0–14, 15–24, 25–44, 45–64, 65+), violence mechanism was grouped into (unarmed brawl, assault with object, stabbing, firearm and other), injury type as (penetrating versus non-penetrating) and ISS as (1–14 vs 16–75)

The reference group for each model outcome was the group not having that specific characteristic

## Discussion

The outcomes from this study provide evidence suggesting that ethnicity of violence-related casualties influence the mechanism, circumstances and severity of injury, and consequently hospital resource utilization.

The evidence shows that the mechanism of injury varies by ethnicity, IBE are at greater risk of hospitalization due to stab injuries, IBFSU for unarmed brawl injuries and AI for firearm injuries. Both AI and IBE are also at a greater risk of hospitalization due to injuries by assault with an object. These findings may be partially associated with the age and gender distributions in each population group. In comparison to IBFSU and AOI, IBE and AI males and young adults, aged 15–44 years, are at greater risk for violence-related hospitalizations. The younger age and male gender characteristics of IBE and AI casualties may contribute to their increased risk for injuries due to stabbings and fire arms as supported by our observation in adjusted analyses, and consistent with the available evidence reporting that violence by sharp objects and firearm is more prevalent in these segments of population [42]. Attributed to masculinity and gender values, younger people and males are more apt to risky behaviors and thus may have greater chance of being involved in violence in general, and in more severe forms of violence in particular [2, 3, 43–46].

In addition to injury mechanism, differences in place and time of event were observed. The street was a common place of injury for the immigrant groups, IBE and IBFSU. While among all groups the majority of hospitalizations occurred on the weekend or at night, the percentage was even greater among the Ethiopian immigrants. These outcomes may indicate that immigration has influential effects on where injury events occur. This information should be used by policy makers to develop interventions which focus on the peak times and places of violence-related injury events. It can also be likely that the injury setting characteristics may contribute to the observed differences in violence mechanism between the population groups, which were demonstrated in the adjusted analyses. The comparison population groups had a higher risk of hospitalization due to specific types of violence, even after controlling for possible confounding factors, implying that ethnicity is an independent factor.

It is possible that situational factors such as ease of access to weapons may play a role in determining violence and its consequences [47], although this study has not investigated these aspects. There is evidence suggesting that practices of carrying and using knives in Israel are growing [1, 21], since they are easily available. There is also evidence reporting that pupils from low socioeconomic status (SES) families have higher levels of weapon carrying [48]. The availability of weapons and the act of carrying them are risk factors for violence. The high prevalence of carrying weapons, such as knives, and using them in Israel [1, 21] can be speculated to be among the factors associated with increased rates of stab-related injuries, warranting the importance of intensifying control measures against access to and carrying of such weapons. The findings presented identify IBE as a distinct high risk group and their needs must be addressed. The availability of firearms is a major determinant of their use and influence on homicide rates [1, 2, 46]. Surprisingly, even the possession of licensed firearms has been reported to affect the risk of violence-related injuries [17]. People with firearm possession are more likely to endorse aggressive attitudes that increase their risk for retaliatory violence [49]. In Israel, there are increasing media reports of illegal gun possession and armed crimes, particularly in Arab towns. It has been reported that firearms are easy to obtain, that is, both makeshift pistols which are being manufactured in workshops in Arab villages or in the Palestinian territories, and weapons which are often smuggled or stolen. There are also social media reports of increasing gun carrying to school by children in the Arab sector [22]. Accordingly, all such factors may increase risk of firearm injuries among Arabs, which is supported by our finding. The international literature also has shown evidence that stab and firearm injuries are more common among ethnic minorities, similar to our findings of higher risks among the minority groups in Israel [3–5, 7, 46].

The differences in injury type between the population groups can be attributed to their differences in the involved violence mechanism. For example, the frequency of a combination of firearm and stab injuries was highest among AI, which will explain for the greater rate of penetrating injuries in this population group. Israel has a lower rate of personal gun ownership, stricter gun control laws, and its policy discourages personal gun ownership [50]. Our findings, however, may highlight the importance of intensifying regulatory and monitoring activities on firearms. In an effort to reduce firearm-related injuries, there may be an urgent need to enforce the illegal access and distribution of firearms, especially by theft or unlawful sales, in addition to enhancing efforts in restricting weapons in certain settings, for example, leisure facilities, public places and school premises, as such interventions are proven to be effective in reducing violent injuries [42, 43]. In addition, due to the ethnic differences, formulating population-specific and socio-culturally appropriate violence prevention and intervention programs is crucial.

In comparison to the general Israeli population, the three minority groups studied, IBE, AI and IBFSU appear to be disproportionately affected by violence-related injuries, as demonstrated by the greater proportion of IBE, AI and IBFSU casualties from the overall violence casualties than their respective share in the general Israeli population; 3.2 times greater for IBE, 2.2 times greater for AI and 1.6 greater for IBFSU. These disparities can be speculated to be due to differential exposure to risk and protective factors. It is known that there is no single reason explaining why some populations are more vulnerable to injury in general, and specifically to different types of violence-related injuries. Nevertheless, these differences in violence injuries between the ethnic groups compared in our study may be, at least in part, be a reflection of the many socioeconomic differences. There is evidence that the SES of minority groups in Israel, in particular the Ethiopian community and Arabs, is lower than that of the other population groups and they often reside in disadvantaged neighborhoods. The Ethiopian community has the lowest SES amongst all population groups in the country [25–28]. A large body of evidence shows an association between lower SES and an increased risk of sustaining violence-related injuries [1, 2, 20, 30–32, 51–55]. Low income, low education level, unemployment, unskilled labor and job dissatisfaction, poverty, low social standing and integration difficulties may be speculated to be associated with behavioral changes, which may increase exposure and susceptibility to violence and worse outcomes. Socioeconomic disparities may be associated with various risky behaviors, including substance abuse and crime, which may increase risk of violence-related injuries [6, 17, 30–32]. In addition, there may be low negotiation skills attributable to lower education level, and as a result unprecedented situations may end up with events that can lead to injury, in particular to the more severe forms of violence. Cultural practices and social values may play roles in experiencing inequalities in violence. Violence may be regarded as a practical option to conflict resolution [42, 56]. Contextual factors in disadvantaged neighborhoods may create suitable situations to exposure and susceptibility to violence, as in such neighborhoods there may be high rates of crimes, illicit drugs dealing, gang membership and deviant peer groups [8, 31, 57, 58].

In addition to changes in the socioeconomic and professional status, the immigration process, which includes obstacles, such as language barrier and cultural differences, may hinder integration of immigrants into the Israeli society and lead to violence-related behaviors, which may contribute to violence injuries among immigrants from Ethiopia and the former Soviet Union [17, 59]. It is challenging for immigrants to adapt between their cultures of the past and fitting into the new culture, the Israeli society. These clashes often play a crucial role in violent behavior and consequently on injuries [11, 60]. Furthermore, new immigrants and minority groups may experience discrimination leading to violence-related behaviors [25, 61]. Due to language and cultural barriers, access to and utilization of preventive services among Ethiopian immigrants may also be lacking.

While there were significant differences in hospital resource utilization, as a function of injury severity, no difference in in-hospital mortality was found. This finding could be a result of appropriate trauma care to all population groups, without any discrimination. In addition, it shows the efficacy of the Israeli trauma care system and the national health insurance law, which provides equal quality of care for all Israeli residents regardless of ethnicity, gender or country of origin where fees are not prerequisite for receiving care [33, 37, 38].

### Limitations

First, the study population includes victims of violence, and not necessarily the perpetrators. Thus, we are unable to identify the victim-perpetrator relationship, such as stranger, friend, family member, or intimate partner, or other demographic characteristic.

Second, the study included only the first generation of immigrants from Ethiopia or former Soviet Union, that is, those born abroad. Although identifying the immigration effect on the next generation is important, the current trauma registry database does not provide the parents’ country of origin, which can be considered an important limitation.

A third limitation originates from the inclusion criteria of the ITR. The ITR does not include mortality prior to arriving at the medical center. In addition, casualties with minor injuries, who were not hospitalized, were not included in this study, which might have resulted in selection biased estimate. Since the majority of hospitalized trauma patients and almost all severely wounded patients are treated in the ITR participating hospitals, we can conclude that this study provides plentiful, representative and valuable information in understanding violence injury characteristics and outcomes in Israel.

Fourth, since the trauma registry does not include information on socio-economic position, its potential influence could not be investigated. We recommend future research to explore the contribution of socioeconomic position on violence-related injuries among different population groups.

## Conclusions

This paper highlights that ethnicity of violence-related casualties differs by the type of violence, circumstances of injury, injury severity and consequently by hospital resource utilization. The outcomes should be addressed by formulating and implementing population-specific and socio-culturally appropriate violence prevention and response programs.

### Policy implications

The findings of this study highlight the differences between populations groups living in the same country with regard to the specific attribute of violence-related injuries. In addition, the differential rates of violence injuries, and in particular those of severity of injuries may indicate that the populations may suffer differently from worse health, behavioral and socioeconomic consequences [43, 62]. Building on our assessment, we propose that violence-related injury prevention and response efforts targeting IBE should focus on stabbing-related injuries and to those injuries occurring on streets and during weekends/weeknights; whereas among AI firearm injuries need to be addressed; and among IBFSU, interventions need to focus on preventing unarmed brawl injuries, especially focusing on street fights. Violence prevention efforts need to be tailored to at-risk population groups and to integrate problem-solving approaches. Hot spots and neighborhoods as well as peak times for violence need to be identified. Integrating law enforcement with community and problem-oriented policing in an effort to deal with changing cultural and social norms that support violence may be effective in achieving violence prevention. Local and socio-cultural differences need to be considered and all relevant partners involved during the planning and implementation of violence prevention and response programs. In addition, existing legislations and policies regarding firearms should be reviewed.

In Israel, there are a variety of programs operated aimed at preventing violence and crime across the country, including City Without Violence, Metzila - Community and Crime Prevention Division, Parent Patrols, the Anti-Drug and Alcohol Authority, Domestic Violence - Prevention and Treatment, and the police high school program, among others. There are also mentions of violence prevention efforts with a focus on unique demographic groups such as Israelis of Ethiopian descent, Arabs, and other segments of the population as per their unique characteristics [63]. Although national laws, action plans, policies and programs relevant to several type of violence are available in the country [43], their enforcement and implementation may not be adequate on levels necessary to achieve reductions in violence inequalities. In the wake of our results, the Israel government may need to be aware of the differences in mechanism and circumstances of violence injuries as well as hospitalization characteristics between populations of different ethnicity and country of origin, thus to allocate adequate financial, material and human resources in the efforts to support violence prevention, response and research activities so as to reduce the inequalities. Hospitals need to anticipate ethnic variation in injury pattern and severity and hence that of hospital resource utilization so as to optimize patient outcome and operational efficiency following violence injury. In addition, health care facilities can be important partners in violence prevention activities through several roles, such as increasing awareness of the problem and; serving as educators, trainers, consultants, advocates or coordinators in joint community-based activities; and actively participating with ministry of health in data collection and reporting as well as in research activities [62]. The educational system can jointly work with relevant partners in identifying, handling and referring individuals affected by or at risk for violence, as well as in research activities. Prevention efforts need to be fully integrated into clinical and community settings. Last but not least, further research studies need to be carried out to evaluate the underlying factors for the disparities in violence between the population groups, particularly in violence type and circumstances as well as impacts of violence and victim-perpetrator relationship.

### What the present study adds to existing knowledge?

Populations of different ethnicity and country of origin differ by the type of violence-related injury circumstances and characteristics, and consequently by hospital resource utilization.

- In comparison to AOI, immigrants (IBE and IBFSU) and minorities (AI) are disproportionately affected by violence-related injuries.
- While stab injuries were most common among IBE, firearm injuries were greatest among AI and unarmed brawl-related injuries were greatest among IBFSU.
- Injury events among immigrants were most likely to occur on the street/road.
- Violence-related injury events were most frequent on weekends and weeknights, especially among IBE.
- Injuries to head and face were more prevalent among IBE and IBFSU, while injuries to extremities were more frequent among AI.
- Violence-related injury severity and hospital utilization was greatest among IBE, in comparison to the other population groups.
